# PID/WAG-mediated phosphorylation of the *Arabidopsis* PIN3 auxin transporter mediates polarity switches during gravitropism

**DOI:** 10.1038/s41598-018-28188-1

**Published:** 2018-07-06

**Authors:** Peter Grones, Melinda Abas, Jakub Hajný, Angharad Jones, Sascha Waidmann, Jürgen Kleine-Vehn, Jiří Friml

**Affiliations:** 1 Institute of Science and Technology Austria (IST Austria), Am Campus 1, 3400 Klosterneuburg, Austria; 2 Laboratory of Growth Regulators, Palacký University, Křížkovského 511/8, 771 47 Olomouc, Czech Republic; 3 Department of Applied Genetics and Cell Biology, University of Natural Resources and Life Sciences (BOKU), Muthgasse 18, 1190 Vienna, Austria; 4 Cardiff School of Biosciences, The Sir Martin Evans Building, Museum Avenue, Cardiff, CF10 3AX United Kingdom

## Abstract

Intercellular distribution of the plant hormone auxin largely depends on the polar subcellular distribution of the plasma membrane PIN-FORMED (PIN) auxin transporters. PIN polarity switches in response to different developmental and environmental signals have been shown to redirect auxin fluxes mediating certain developmental responses. PIN phosphorylation at different sites and by different kinases is crucial for PIN function. Here we investigate the role of PIN phosphorylation during gravitropic response. Loss- and gain-of-function mutants in PINOID and related kinases but not in D6PK kinase as well as mutations mimicking constitutive dephosphorylated or phosphorylated status of two clusters of predicted phosphorylation sites partially disrupted PIN3 phosphorylation and caused defects in gravitropic bending in roots and hypocotyls. In particular, they impacted PIN3 polarity rearrangements in response to gravity and during feed-back regulation by auxin itself. Thus PIN phosphorylation, besides regulating transport activity and apical-basal targeting, is also important for the rapid polarity switches in response to environmental and endogenous signals.

## Introduction

The plant hormone auxin, indole-3-acetic acid (IAA), controls plant growth and development by modulating fundamental cellular processes such as cell division, expansion, and differentiation^1^. Intercellular auxin transport and metabolism are responsible for changes in cellular auxin concentration^2–4^, which leads to different auxin responses^5–7^. In recent years, detailed characterization of auxin transport proteins and their regulators has broadened our knowledge of polar auxin transport, auxin gradient formation and mechanisms of differential growth and organogenesis^8,9^. The most prominent auxin transporters are AUX1/LIKE AUX1 auxin importers^10,11^, P-glycoproteins of the ATP-binding cassette transporter family of auxin exporters^12^, and polarly localized PIN auxin exporters^4,13^. Although all of these proteins are involved in passing auxin across the plasma membrane out of or into the cell, it seems that PIN auxin efflux carriers^14^ are predominant in mediating the directionality of the intercellular auxin flow by virtue of their polar subcellular localization in auxin-transporting cells^15,16^.

One of the major regulatory mechanisms of PIN polar targeting is phosphorylation. Several studies demonstrated that serine/threonine protein kinases from the AGCVIII kinase family phosphorylate the hydrophilic loop of PIN proteins, which correlates with the change in PIN polar localization^16–21^. Three of these kinases, PINOID (PID), WAG1 and WAG2^22–24^ play a crucial role. They are at least partially functionally redundant^18,24,25^. Overexpression of these kinases leads to a basal-to-apical (rootward-to-shootward) shift in PIN polarity that causes disruption of the auxin maxima and to the collapse of the root meristem and agravitropic root growth^22,23^. On the other hand, *pid, wag1, wag2* single or multiple mutants show more preferential basal PIN localization causing deprivation of auxin from the shoot meristem and a pin-like inflorescence phenotype or more basal localization of the otherwise apically localized PIN2 in root epidermis leading to agravitropic root growth^18,23,26^. In accordance with the model that more PID-dependent phosphorylation leads to a preferentially apical PIN localization, phosphomimicking or phosphodead mutations of serine/threonine amino acids within the PIN hydrophilic loop show more apical or basal localization respectively.

Recently, a related subfamily of AGCVIII kinases involved in auxin transport and plant development has been identified. D6 protein kinase (D6PK) localizes to the basal membrane of *Arabidopsis* cells in root and co-localizes with several PIN proteins such as PIN1, PIN2 and PIN4. It was shown that D6PK can interact directly with PIN1 protein and phosphorylate it^27,28^.

Changes in polar subcellular localization of PINs seem to be an essential mechanism for redirecting auxin fluxes in response to different environmental stimuli. For example, the apolar distribution of PIN3 becomes polar after gravitropic stimuli, leading to relocalization of PIN3 towards the gravity vector and correlating with changed auxin fluxes^5,29,30^. A similar phenomenon of PIN3 relocation, albeit slower, has been observed during the hypocotyl phototropic response^31^. Recently, a subsequent second re-polarization event during hypocotyl bending has been identified, which is important for resetting the asymmetry in the PIN polar distribution; ultimately leading to the termination of the bending^32^. This second repolarization is likely related to the auxin feed-back on PIN polarity as seen in so called auxin canalization processes of leaf venation and vascular tissue regeneration^33,34^. Although PIN3 and also PIN7^35–37^ relocalization events are likely to be involved in redirecting auxin fluxes to create particular growth responses, insight into the underlying mechanism of relocation is still limited.

In this study we examined potential phosphorylation sites in the PIN3 hydrophilic loop and their role in tropic responses. We identified sites that play a role in both PIN3 polarity rearrangements during gravitropic responses, thus demonstrating a crucial role for PIN phosphorylation in polarity switches in response to external signals such as gravity or endogenous signals such as auxin itself.

## Results

### Importance of phosphorylation for gravity-mediated PIN3 relocation and bending

**Figure 1:**
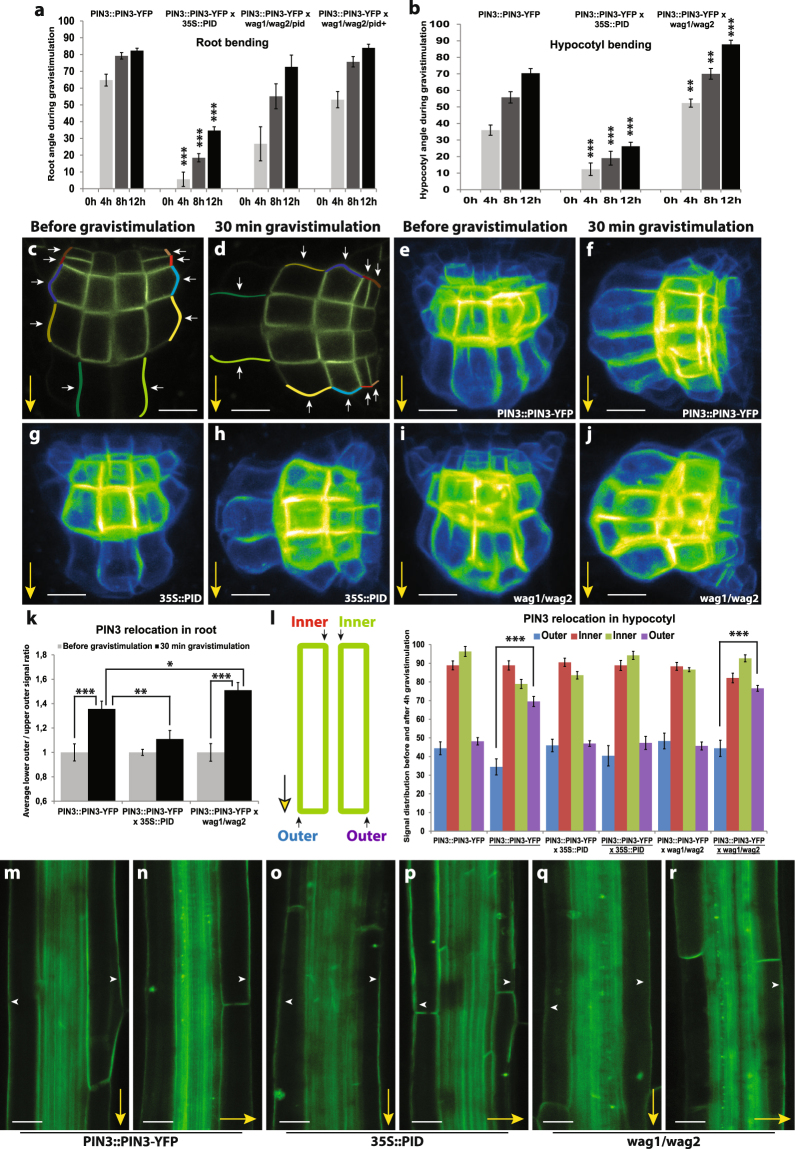
PID kinase is involved in the gravitropism and gravity-induced PIN3 relocation in root and hypocotyl. (**a**) Root bending assay in wild-type, *35S::PID*, *wag1/wag2/pid* and *wag1/wag2* double mutants from the *wag1/wag2/pid*+ population after gravistimulation. (**b**) Hypocotyl bending assay in in wild-type, *35S::PID* and *wag1/wag2* backgrounds after gravistimulation. Student’s T-tests were calculated for the comparison of each time point with the control (*PIN3::PIN3-YFP*). In hypocotyl, *35S::PID* shows less and *wag1/wag2* more gravitropic bending as compared to the control, while only roots of *35S::PID* exhibited slower bending after gravistimulation. (**c**,**d**) Schemes representing cellular membranes used for quantification of PIN3-YFP protein relocation in root columella cells. Signal intensity ratio before and after gravistimulation was calculated between lower outer (light colors) and upper outer (dark colors). Signal ratio for one root was calculated as an average of signal intensity ratios. (**e–j**) PIN3-YFP relocation in columella before (**e**,**g**,**i**) and after 30 minutes of gravitropic stimulation (**f**,**h**,**j**) in wild type, *35S::PID* and *wag1/wag2*. (**k**) Quantification of gravity-mediated PIN3-YFP relocalization in root. Signal before gravistimulation was normalized to 1. Student’s T-tests were calculated for the comparison of each time point with the control (*s*). *35S::PID* shows less and *wag1/wag2* more gravity-induced PIN3 relocation. (**l**) Scheme representing membranes used for quantification of PIN3-YFP protein relocation in hypocotyl endodermal cells and quantification of gravity-mediated PIN3-YFP relocalization in hypocotyls. Underlined genotypes represent samples after gravistimulation. Student’s T-tests were calculated for the comparison of outer membranes signal within each line. *35S::PID* shows less gravity-induced PIN3 relocation in hypocotyl. (**m–r**) PIN3-YFP protein localization in hypocotyl before (m,o,q) and after 4 hours of gravitropic stimulation (n,p,r) in wild type, *35S::PID* and *wag1/wag2*. Experiments were repeated 3 times with 10–15 roots or hypocotyls per sample. Arrowheads indicate localization of the PIN3 protein. Error bars represent SE, (*p < 0.05, **p < 0.01, ***p < 0.001). Yellow arrows indicate gravity vector. Bars = 10 µm.

Gravistimulation has been shown to induce changes in polar PIN3 localization in roots where PIN3 relocates towards the bottom side of the columella cells after the gravitropic stimulus^5,29,35–37^. PIN phosphorylation by PID has an impact on PIN polarity and auxin transport directionality in different developmental contexts by regulating apical-basal PIN localization^16,17,19^. We tested whether PID contributes to the gravitropic response and also to the gravity-induced relocation of PIN3 in roots and hypocotyls. It is known^18,22^ that root bending in PID overexpressing line (*35S::PID*) is defective (Fig. 1a). As analyzed previously^38^, *wag1/wag2/pid* triple mutant exhibited slightly slower root bending compared to control, while *wag1/wag2* double mutant from the *wag1/wag2/pid*+ segregating population showed no difference in root bending after gravitropic stimuli compared to control (Fig. 1a). On the other hand, during hypocotyl bending, *35S::PID* line showed slower whereas *wag1/wag2* double mutant faster gravitropic response (Fig. 1b).

Overexpression or lack of WAG1/WAG2/PID has been shown to disturb PIN1 and PIN2 localizations^19^, which can explain defects in gravitropism. We were interested if gravity-induced PIN3 relocation was also affected, thus we examined PIN3 distribution in root columella cells following gravitropic stimulation. We quantified changes by comparing PIN3-YFP signal intensity on the upper outer and lower outer sides of columella cells (Fig. 1c,d). After 30 minutes of gravistimulation, PIN3 relocates to the new basal cell sides following gravity (Fig. 1e,f,k). In the *35S::PID* overexpression line we observed a significant reduction in this PIN3 polarization (Fig. 1g,h,k). In a similar way, we observed that the *wag1/wag2* double mutant exhibited a more pronounced PIN3-YFP relocation rate after gravitropic stimuli (Fig. 1i–k). Also in the hypocotyl, phosphorylation by PID plays an important role in PIN3 polarization during the tropic response^30,31^. After 120 minutes of gravistimulation we observed relocation of PIN3-YFP to the bottom side of endodermal cells (Fig. 1l–n). Analogously to roots, overexpression of PID in the *35S::PID* line caused a decrease in the relocation rate of PIN3-YFP after gravistimulation (Fig. 1l,o,p) whereas in *wag1/wag2* the PIN3 relocation was more pronounced (Fig. 1l,q,r). Thus, the rates of PIN3 relocalisation in the *PID* overexpressor correlate with the decreased rates of bending observed in these plants.

Further, we tested the potential involvement of PIN phosphorylation by D6PK kinase^28^ during the root gravitropic response. After 12 hours of gravitropic treatment we observed no significant differences in any *d6pk* mutants or overexpression lines (Figure S1a), agreeing with previously reported mild gravitropic defects^28^. This argues against an important role of D6PK in root gravitropism, and suggests that the contribution of D6PK for the regulation of PIN activity, either in columella cells for redirection of auxin fluxes, or in epidermis for shootward auxin transport, is not crucial.

Overall these results show that PID/WAG, but not D6PK, play an important role in gravity-mediated PIN3 repolarisation and in gravitropic bending both in roots and shoots. Higher PID expression inhibits PIN3 polarization and gravitropic bending whereas decreased PID/WAG expression leads to increased PIN3 polarization.

### Putative phosphorylation residues in the PIN3 hydrophilic loop

**Figure 2:**
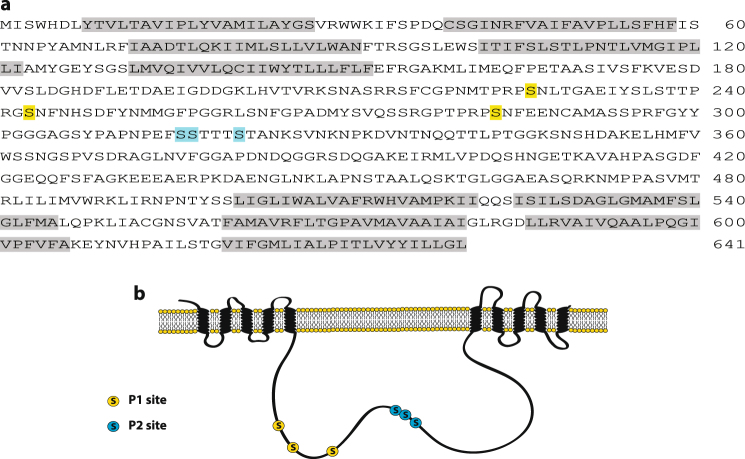
Phosphorylation sites in PIN3. (**a**) Positions of mutated amino acids in the sequence of PIN3 protein. P1 site is marked in yellow and P2 in blue. Transmembrane domains are highlighted in grey. (**b**) Scheme representing positions of mutated residues within PIN3 protein.

Previous studies showed that PID directly phosphorylates the central hydrophilic loop of PIN proteins both *in vitro* and *in vivo*^17,18,28^. Therefore, we investigated the putative phosphorylation sites in the PIN3 loop. Two putative phosphorylation clusters, P1 and P2, both containing three serines (P1: S226, S243, and S283; P2: S316, S317, and S321) (Fig. 2a,b), were chosen. The P1 sites are the previously described conserved TPRxS motif^18,19^, whereas the P2 sites are analogous to a described PIN1 phosphorylation site^16,18^ that plays an important role during the basal-to-apical PIN1 relocalization^16^. Four different PIN3 mutant constructs were prepared, in which serines were either substituted by alanines (P1A: S226A, S243A, and S283A; P2A: S316A, S317A, and S321A) to mimic the non-phosphorylated state or by aspartic acid to mimic the constitutively phosphorylated status (P1D: S226D, S243D, and S283D; P2D: S316D, S317D, and S321D).

All mutant variants were cloned under the control of the native promoter and introduced into the wild type and *pin3–4* mutant to evaluate their impact on PIN3 function. The transformed plants did not exhibit any strong and obvious developmental defects. Levels of mutated PIN3 transcript in plants were evaluated by qPCR and only lines exhibiting similar expression level as PIN3 in wild type were used for further experiments (Fig. S1b). None of the phosphomutant variants exhibited defects in root length or meristem size, but all of them showed a slight reduction in the hypocotyl length in dark-grown seedlings (Fig. S1c,d,g). In two mutant variants PIN3-YFP-P1D and PIN3-YFP-P2D, the number of emerged lateral roots was slightly lower than that of the wild type (Fig. S1e). Analysis of lateral root stages revealed an increased number of first-stage primordia in PIN3-YFP-P2A and PIN3-YFP-P2D mutant variant (Fig. S1f), whereas the other mutant variants did not reveal any defects during lateral root formation. This suggests some contribution of PIN3 phosphorylation at our chosen sites during root and hypocotyl growth, in particular during the first stages of lateral root development. However, the effects of the chosen phosphomutations on overall development were not very prominent, suggesting that these sites are not important for an overall PIN3 activity, but potentially for some more specific aspects of its function.

### *In vivo* phosphorylation of PIN3 mutant variants

**Figure 3:**
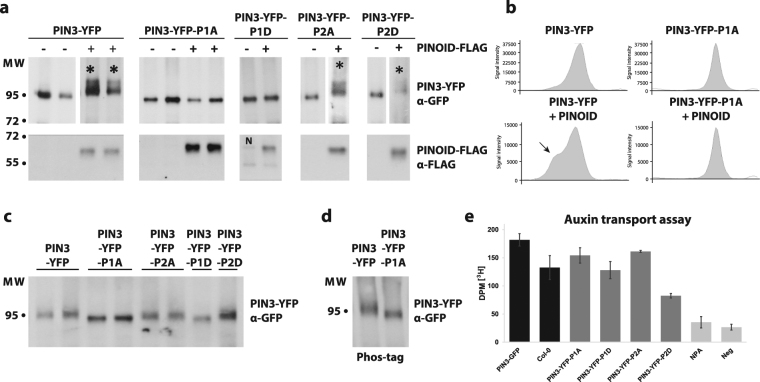
*In vivo* phosphorylation of PIN3-YFP and mutant variants. (**a**) Transiently expressed PIN3-YFP and all mutant variants in *N. benthamiana* with or without co-infiltration with PINOID-FLAG. Total protein was extracted, separated by SDS-PAGE, blotted and probed with anti-GFP and anti-FLAG. Phosphorylation of PIN3-YFP, PIN3-YFP-P2A and PIN3-YFP-P2D by PINOID appears as a distinct smear above the main band (marked with star). Results from one single blot are shown, some lines were exposed longer due to weaker signals. The complete blots and Ponceau stain are shown in Figure S3. N indicates carryover signal from anti-GFP (breakdown product of PIN3-YFP-P1D). (**b**) Lane profiles of the anti-GFP blots for PIN3-YFP and PIN3-YFP-P1A with or without PINOID from (a). Arrow marks additional peak representing phosphorylated protein. (**c**) Faster migration of PIN3-YFP-P1A and PIN3-YFP-P1D in SDS-PAGE compared to WT and PIN3-YFP-P2A/D (**d**) Phostag gel revealed no enhancement in the differences of the migration between PIN3-YFP and PIN3-YFP-P1A. (**e**) Rootward transport of radiolabeled ^3^H-IAA in decapitated hypocotyls. Neg represents negative control for diffusion in agar. Treatment of 10 μM N-1-naphthylphthalamic acid (NPA), an auxin transport inhibitor, was used as additional negative control. Student’s T-test was calculated for the comparison of each line with the control (Col-0). Error bars represent SE.

Previous study has shown by mass spectrometry that PIN3 protein is phosphorylated *in vivo* at multiple sites, including the P1 and P2 sites^39^. We evaluated the contribution of the P1 and P2 sites to total phosphorylation of PIN3 by PID kinase. PIN3::PIN3-YFP and all of the mutant constructs were transiently expressed in *N. benthamiana* leaves via *Agrobacterium* infiltration, with or without co-infiltration with PID. Co-expression of PID induced a shift in PIN3-YFP, indicating phosphorylation (Fig. 3a,b). A similar PID-dependent shift was observed when co-expressed with PIN3-YFP-P2A and PIN3-YFP-P2D mutant variants, but not with PIN3-YFP-P1A or PIN3-YFP-P1D mutant variants (Fig. 3a,b; Figure S2a). In fact, even without PID co-expression, the PIN3-YFP-P1A and PIN3-YFP-P1D proteins migrated faster in the SDS-PAGE gel, with less diffusion (sharper bands) compared to the WT or PIN3-YFP-P2A/D mutant variants (Fig. 3c). Since the altered migration and appearance on SDS-PAGE of the PIN3-YFP-P1A and PIN3-YFP-P1D mutant proteins may reflect decreased phosphorylation by endogenous *N. benthamiana* kinases, we ran the same samples on Phostag gels, but no major enhancement of the difference in migration between PIN3-YFP and PIN3-YFP-P1A/D was observed (Fig. 3d). This suggests that the difference in migration is not caused by less endogenous phosphorylation of the P1 site mutant variants. Overall, these results indicated that the PID can still phosphorylate PIN3 when the P2 sites are mutated, while mutations in P1 site largely abolished phosphorylation of PIN3 protein by PID kinase.

Next, we evaluated whether mutations in phosphorylation sites might affect auxin transport activity of the PIN3 mutant variants. Etiolated hypocotyls were decapitated and used to measure ^3^H-IAA transported basipetally into the hypocotyl. We observed altered transport rates in both phosphomimic variants, particularly in PIN3-YFP-P2D compared to the wild type (Fig. 3e). This suggests that the selected phosphorylation sites in PIN3 protein are important for the directional auxin transport in the hypocotyl, notwithstanding that the major contributor of the hypocotyl basipetal/downward transport is believed to be the PIN1 auxin transporter^4^.

### PIN3 phosphorylation in the root gravitropic responses

**Figure 4:**
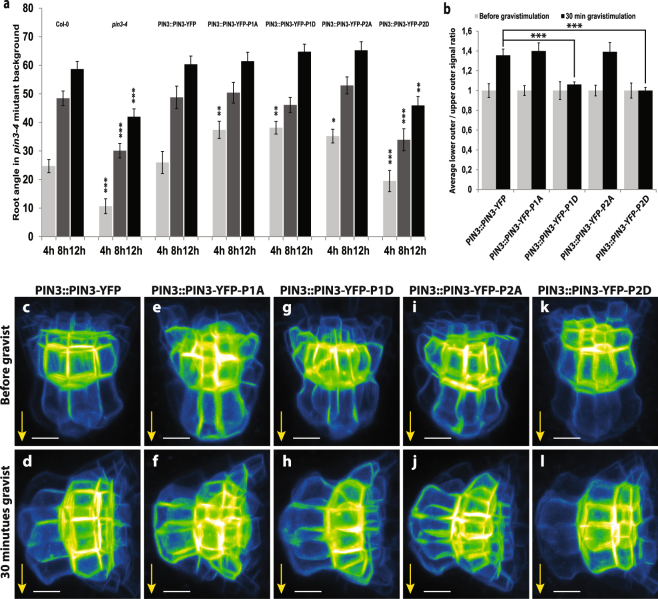
PIN3 phosphorylation is required for PIN3 polarization and root gravitropic response. (**a**) Root bending kinetics of PIN3 mutant variants during gravitropic response. Root curvatures were measured every 4 hours. Student’s T-test was calculated for the comparison of each line with the control (Col-0). PIN3-YFP-P2D mutant variant shows slower root bending. (**b**) Quantification of PIN3 polarization in columella cells before and after gravistimulation. Signal before gravistimulation was normalized to 1. Student’s T-test was calculated for the comparison of each line with the control (*PIN3::PIN3-YFP*). Both phosphomimic variants, PIN3-P1D and PIN3-P2D, exhibit less gravity-induced PIN3 relocation. (**c–l**) Localization of PIN3-YFP (**c**,**d**), PIN3-YFP-P1A (**e**,**f**), PIN3-YFP-P1D (**g**,**h**), PIN3-YFP-P2A (**i**,**j**), and PIN3-YFP-P2D (**k**,**l**) before (upper row) and after 30 minutes (lower row) of gravitropic stimulation in the wild type background. Values are the average of three biological replicates (n > 10 per time point on each replicate). Error bars represent SE, (*p < 0.05, **p < 0.01, ***p < 0.001). Yellow arrows indicate gravity vector. Bars = 10 µm.

We tested the effect of the PIN3 phosphomutant variants on gravitropic response and gravity-induced PIN3 relocalization in roots. All phosphomutant variants introduced in the *pin3-4* background partially rescued the *pin3* mutant phenotype. During the gravitropic response, PIN3-YFP-P1A, PIN3-YFP-P1D and PIN3-YFP-P2A were bending slightly faster (after 4 hours) as compared to the wild-type, but the PIN3-YFP-P2D mutant variant exhibited the least rescue showing defective gravitropic bending comparable to the *pin3-4* mutant (Fig. 4a). Thus the PIN3 phosphomutant variants were not able to completely complement the wild type PIN3 function in root gravitropism.

At the cellular level, all PIN3 phosphomutant variants showed the apolar distribution of the PIN3 protein in columella cells in non-stimulated roots similar to the wild type (Fig. 4c,e,g,i,k). After gravity stimulation (30 min), the PIN3-YFP protein relocated to the new lower sides of columella cells (Fig. 4b–d). Phosphodead PIN3-YFP-P1A and PIN3-YFP-P2A exhibited similar relocation rate to wild type (Fig. 4b,e,f,i,j), whereas phosphomimic PIN3-YFP-P1D and PIN3-YFP-P2D variants exhibited a clear defect in the PIN3 relocation (Fig. 4b,g,h,k,l).

Together, the results suggest the importance of PIN3 phosphorylation in the root gravitropic response, in particular that mimicking constitutive phosphorylation inhibits gravity-induced PIN3 relocation similar to the effects observed in *35S::PID* lines.

### PIN3 phosphorylation in the hypocotyl gravitropic response

Next, we tested the effect of the PIN3 phosphomutant variants on the gravitropic response and gravity-induced PIN3 relocalization in hypocotyls. Gravistimulation induces PIN3 polarization towards the new bottom sides of endodermal cells leading to auxin accumulation at the lower side of hypocotyl. This auxin accumulation leads to a second, subsequent PIN3 polarization, during which PIN3 in cells of the lower hypocotyl sides polarizes back to the upper cell sides, restoring PIN3 expression symmetry and aiding termination of bending^30,32^.

**Figure 5:**
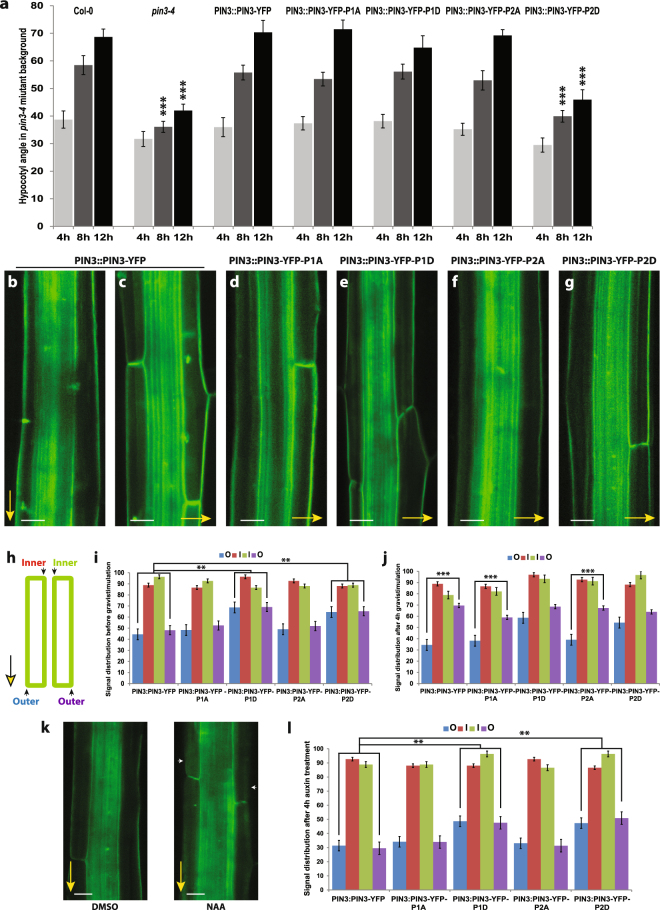
PIN3 phosphorylation is required for PIN3 polarization and hypocotyl gravitropic response. (**a**) Hypocotyl bending kinetics of PIN3 mutant variants during gravitropic response. Curvatures were measured every 4 hours. Student’s T-test was calculated for the comparison of each line with the control (Col-0). PIN3-P2D mutant variant shows slower hypocotyl bending. (**b–g**) Localization of PIN3-YFP before and after 4 hours of gravistimulation (**b**,**c**) and after gravistimulation in PIN3-YFP-P1A (**d**), PIN3-YFP-P1D (**e**), PIN3-YFP-P2A (**f**), and PIN3-YFP-P2D (**g**). Yellow arrows indicate gravity vector. (**h**) Scheme of quantification showing measured membranes in hypocotyl. (**i**) Quantification of PIN3 signal distribution in hypocotyl endodermal cells. Student’s T-test was calculated for the comparison of outer membranes signal within each line. PIN3-P1D and PIN3-P2D exhibit higher signal intensity on outer-lateral membranes. (**j**) Quantitative evaluation of gravity-dependent PIN3 relocation in hypocotyl endodermal cells. Student’s T-test was calculated for the comparison of outer membranes signal within each line. Both PIN3-P1D and PIN3-P2D show defective gravity-induced PIN3-YFP relocation. (**k**) Localization of PIN3-YFP before and after 4 hours of 10 μM NAA treatment in wild type. White arrows indicate depletion of PIN3 protein from outer-lateral cell membranes. (**l**) Quantification of PIN3-YFP signal in endodermal cells of hypocotyl after 4 hours of 10 μM NAA treatment. Student’s T-test was calculated for the comparison of each line with the control (*PIN3::PIN3-YFP*). PIN3-P1D and PIN3-P2D exhibit reduced auxin-induced PIN3 inner-lateralization. Error bars represent SE, (**p < 0.01, ***p < 0.001). Yellow arrows indicate gravity vector. Bars = 10 µm.

Similar to the situation in the root, the line expressing PIN3-YFP-P2D showed a weakest rescue of hypocotyl gravitropism (Fig. 5a). At the cellular level, we observed enhanced signal intensity in the outer lateral membranes of the endodermal cells in the phosphomimicking PIN3-YFP-P1D and PIN3-YFP-P2D lines when compared to the control (Figs 5i, S2b,d,f). After 4 hours gravistimulation, the PIN3-YFP in the cells of the upper hypocotyl side relocates from the outer/upper lateral to the inner/bottom lateral membranes and at the lower hypocotyl side from the inner/upper lateral to the outer/bottom lateral membranes (Fig. 5b,c,i,j). In phosphodead PIN3-YFP-P1A and PIN3-YFP-P2A mutant variants, this gravity induced relocation was comparable to the wild type PIN3-YFP (Figs 5d,f,i,j, S2c,e). On the other hand, similarly as observed in roots, relocation of the PIN3-YFP protein after gravitropic stimuli was less pronounced in phosphomimic PIN3-YFP-P1D and PIN3-YFP-P2D variants (Figs 5e,g,i,j, S2d,f).

Next we tested the auxin effect on PIN3 polar distribution that has been suggested to be important for feed-back regulation of bending termination. Treatments with natural or synthetic auxins can relocate the PIN3 protein in endodermal cells of hypocotyls from the outer lateral to the inner lateral membranes, the so-called “inner-lateralization”^32^. After 4 hours of NAA treatment we observed a reduced PIN3 inner-lateralization for PIN3-YFP-P1D and PIN3-YFP-P2D phosphomimic variants as compared to the phospho-dead variants or control (Fig. 5k,l).

These data indicate that in both negative gravitropism of the hypocotyl as well as in positive root gravitropism, PIN3 phosphorylation plays a similar role in gravity-induced relocation and gravitropic response in both above and underground organs. In addition, the hypocotyl experiments revealed a role of PIN3 phosphorylation in auxin-induced relocation.

## Discussion

How a directional signal such as gravity is translated within the plant into the directional auxin flow that forms the lateral auxin gradient and thus drives the bending response is conceptually one of the hardest questions in understanding plant tropic responses. The observation that gravistimulation induces changes in the polar localization of PIN auxin transporters, which is consistent with auxin flow being aligned with the gravity vector^5,29,35–37^, provides a possible mechanism for gravity-induced redirection of auxin fluxes. PIN phosphorylation by the protein kinase PID has been implicated in both apical/basal PIN polarity and tropic responses^4,9^.

Here we have demonstrated that PIN phosphorylation and PID/WAGs kinases activity are involved in the gravity-induced relocation of the PIN3 protein in root columella and hypocotyl endodermal cells. Downregulation of PID/WAGs increases gravity-induced PIN3 relocation and consequently gravitropic bending, whereas upregulation of PID reduces PIN3 relocation and bending. To confirm the role of phosphorylation in this process and to assess which potential phosphorylation sites might be important, we chose two clustered sites, P1 (conserved TPRxS motif^18,19^) and P2 (adapted from^16,18^) that have been shown to be phosphorylated *in vivo*^39–41^, and prepared different phosphorylation variants including phosphomimic and phosphodead. Prepared mutant variants in the *pin3-4* mutant background displayed only minor defects in overall development. More specifically, they show defects in the root and hypocotyl gravitropic growth and gravity-induced PIN3 relocation that are in particular observed in the phosphomimic variants. The hypocotyl experiments also revealed a defect in the auxin-mediated PIN3 relocation, again apparent in the phosphomimic variants. These phenotypes of phosphomimic variants correspond with PIN3 relocation and bending phenotypes in root and hypocotyl in *35S::PID* line suggesting that PIN3 phosphorylation at least partly mediated by PID and associated dephosphorylation by phosphatases are required for both gravity-mediated PIN3 relocations and bending.

Our results revealed that the P2 phosphorylation site, which is partially conserved among long PIN proteins^17^, is important for the PIN3-mediated gravitropic responses of roots and hypocotyls. The phenotypes of mutations at this site described here by us are not completely analogous to the one already published for PIN1^16^. Our phosphorylation assays revealed additional phosphorylation sites might co-operate with the P2 site during gravitropic responses. As PID/WAG kinases are not strongly expressed in columella cells^18^, the P2 sites might be a target of MAPK, which may co-operate with PID during phosphorylation-dependent PIN3-mediated gravitropic responses^16,41^.

A similar study about phosphorylation sites in the PIN3 hydrophilic loop has identified a different phosphorylation site, M3 (_209_SNASRRSFCGPNMTPRPS_226_), that is important for subcellular trafficking and PIN3-mediated developmental processes, such as auxin efflux activity, root growth, and root gravitropism^21,42^. Nevertheless, the PIN3-M3 phosphodead mutant variant was demonstrated to be still phosphorylated by PID or WAG1 *in vitro*^21^.

In addition to the role of PID kinase in regulating both PIN transport activity and its polar localization, the related D6PK kinase has been also shown to phosphorylate PIN proteins recognizing overlapping residues as PID kinase, but being more specifically involved in regulating PIN activity^27,28^. Nonetheless, the D6PK involvement in root gravitropic responses is unlikely, as various *d6pk* mutant and overexpression lines exhibit at most insignificant gravitropism defects in the root^28^. In addition, the antagonistic component of PID activity, protein phosphatase 6 (PP6) holoenzyme, also targets and regulates PIN subcellular localization^17,43^. In roots, dephosphorylation by PP6 complex led to changes in PIN polarity from apical to basal^44,45^, thus the role of phosphatases in controlling PIN polar targeting during gravitropic responses should not be ignored. Given the complexity of the number of putative phosphorylation sites in PIN proteins and different kinases and phosphatases, it seems apparent that multiple overlapping PIN phosphorylation mechanisms will be involved in regulation of different aspects of PIN-dependent auxin transport. Nonetheless, our work provides evidence demonstrating that these phosphorylation processes regulate PIN polarity switches and thus auxin fluxes redirections in response to both environmental and endogenous regulations.

### Experimental Procedures

#### Plant materials and growth conditions

The published transgenic and mutant lines were: *PIN3::PIN3-YFP*^46^; *pin3–4* (SALK_005544); *35S::PID-21*^22^; *wag1/wag2/pid*^18^. All seeds were grown on agarose plates containing 1/2 strength Murashige and Skoog medium with 1% sucrose. Seeds were vernalized for 3 days at 4 °C and consequently grown at 18 °C under 16-h-light/8-h-dark photoperiod. For hypocotyl experiments, after stratification the germination was induced by placing the plates in the light for 5–6 hours that were then transferred to darkness and kept at 18 °C for 4 days. For root or hypocotyl gravitropic stimulations, plates with 4-day-old seedlings were turned 90°, scanned at every time point by scanner and the angles were measured by ImageJ. For root length measurements 5 day old seedling were used, for hypocotyl length measurements 4 day old dark grown seedlings. The emerged lateral root assay was performed on 14 day old seedlings and LR primordia were counted with a differential interference contrast microscope BX51 (Olympus). Each experiment was conducted at least in triplicate. For the statistical evaluation, the t-test was done with the Excel statistical package.

### PIN3 phosphorylation mutagenesis

The binary vector pK7m42GW containing PIN3::PIN3-YFP sequence^46^ was used for transgene construction. Four different DNA fragments (PIN3-P1A, PIN3-P1D, PIN3-P2A, PIN3-P2D) possessing different mutations (Table S1) were synthesized with XhoI and AegI restriction sites on the ends. Via classical cloning all four of these fragments were introduced into the PIN3::PIN3-YFP vector. Transformation of these constructs to *Arabidopsis* was accomplished via *Agrobacterium tumefaciens* (strain PMP90)-mediated infiltration by floral dip. All transformed lines were analyzed and at least 3 independent transgenic lines for each construct with similar expression level were used in this study.

### Confocal microscopy

For confocal microscopy, a Zeiss LSM 710 confocal scanning microscope or Zeiss LSM 710 vertical confocal scanning microscope were used. To monitor the gravitropic response, plates were scanned 24 h after gravistimulation. Images were processed in Zeiss ZEN software and ImageJ. Each experiment was performed at least three times.

### Quantitative analysis of PIN3 relocalization in root and hypocotyl

All measurements were performed using ImageJ software (National Institutes of Health; http://rsb.info.nih.gov/ij). Quantification of gravity-induced PIN3-YFP relocalization in columella cells was performed on maximal intensity projections of Z-scans of columella cells by measuring the signal intensity at the apical membranes (marked with dark colors) and comparing with signal intensity of basal membranes (marked with light colors) of the cells on the periphery of columella before and after gravistimulation (see scheme in Fig. 1c,d). Signal ratio for one root was calculated as an average of signal intensity ratios. For quantification of the gravity-induced PIN3-YFP relocalization in hypocotyls single plain images from the same focal plane were taken and the rate of PIN3-YFP fluorescence intensity was compared between the outer PM sides of endodermal cells (see scheme in Figs 1l or 5h). The PIN3 relocation is most clearly visible in the upper endodermal cells, since the lower cell signal is influenced by PIN3-YFP signal in stele. Three replicates of 10–15 seedlings with a synchronized germination start were processed. The presented values are the mean of the averages.

### *In vivo* phosphorylation of PIN3-YFP and mutant variants

PIN3-YFP and mutant variants were transiently expressed in *N. benthamiana* by *Agrobacterium* infiltration, with or without co-infiltration with PID. The same *Agrobacterium* lines were used as those used to generate the transgenic *A.thaliana* plants described above. Since these constructs contained the native AtPIN3 promoter, the infiltrated *N. benthamiana* leaves were treated with 1 µM IAA 24 h before harvesting to promote gene expression, as the AtPIN3 promoter is known to be responsive to IAA^47^. For co-infiltration with PID, we used an inducible PID (pINTAM3-PID^23^) or 35S::PID-FLAG^17^ cloned into pGREEN. pINTAM3-PID was induced with 1 µM 4OH-tamoxifen (Sigma-Aldrich) and 5 µM beta-estradiol for 24 h before harvesting. All infiltrations were performed with p19 to reduce silencing^48^. Samples were extracted based on Abas & Luschnig^49^, with modifications for a quicker extraction in order to detect phosphorylated protein versions. Frozen tissue was ground in liquid nitrogen and collected into extraction buffer containing PhosSTOP (Roche), centrifuged for 3 minutes at 300 g (4 °C) and the supernatant (soluble and membrane fractions) was immediately solubilized with 2% lithium dodecyl sulfate and 10 mM DTE. Samples were centrifuged at 20 000 g for 30 min (4 °C) and the supernatant precipitated by chloroform/methanol^50^. Proteins were separated by SDS-PAGE, blotted and probed with anti-GFP monoclonal mouse antibody (Roche), stripped and probed with anti-FLAG M2 monoclonal mouse antibody (Sigma-Aldrich). As *N. benthamiana* leaves contain a strong non-specific signal from anti-FLAG antibody at about 90 kD, only the lower half of the blot was used for anti-FLAG. For Phos-tag analysis, 25 µM Phos-tag (WAKO) was incorporated into the gel. Phosphorylated bovine casein, ovalbumin, PID and AtPIN1 were used as positive controls for Phos-tag.

### Auxin transport assay

Etiolated seedlings were prepared as described above, except plates were kept at 21 °C for 5 days. Etiolated hypocotyls were decapitated to exclude the effect of auxin biosynthesis in cotyledons and a droplet of AM + agar (1.25%) with ^3^H-IAA (12uL ^3^H-IAA + 10 mL AM + agar) was applied to the apical part of the hypocotyls. After 6 hours, hypocotyls were collected, homogenized in liquid nitrogen and incubated overnight in Opti-Fluor scintillation cocktail (Perkin Elmer). Amount of transported ^3^H-IAA was then measured in a scintillation counter (Hidex 300SL) for 300 s with three technical repetitions. Negative control was performed by inserting the droplet above the decapitated hypocotyl to account for any diffusion though the agar. Additional negative control was performed using 10 μM NPA, to inhibit auxin transport.

### Quantitative qPCR

Total RNA was extracted with the RNeasy kit (QIAGEN). Poly(dT) cDNA was prepared from total RNA with Superscript III (Invitrogen).Quantitative RT-PCR was done with LightCycler 480 SYBR Green I Master reagents (Roche Diagnostics) and a LightCycler 480 Real-Time PCR System (Roche Diagnostics). Data were analyzed with qBASE v1.3.4^51^. Expression levels were normalized to the non-auxin-responsive genes β-TUBULIN (At5g12250), EEF (At1g30230) and CDKA (At3g48750).

## Electronic supplementary material

Supplemental figures and table
