# Supplementary material for: PID/WAG-mediated phosphorylation of the Arabidopsis PIN3 auxin transporter mediates polarity switches during gravitropism
Source: Sci Rep. 2018 Jul 6;8:10279. doi: 10.1038/s41598-018-28188-1 (PMC6035267; doi:10.1038/s41598-018-28188-1)
Supplement: Supplementary file 1 — Supplemental figures and table [file 41598_2018_28188_MOESM1_ESM.pdf]

PID/WAG-mediated phosphorylation of the Arabidopsis PIN3 auxin transporter mediates polarity switches during gravitropism

Peter Grones[1], Melinda Abas[1,3], Jakub Hajný[1,2], Angharad Jones[4], Sascha Waidmann[3], Jürgen Kleine-Vehn[3], and Jiří Friml[1*]

[1] Institute of Science and Technology Austria (IST Austria), Am Campus 1, 3400 Klosterneuburg, Austria

[2] Laboratory of Growth Regulators, Palacký University, Křížkovského 511/8, 771 47 Olomouc, Czech Republic

[3] Department of Applied Genetics and Cell Biology, University of Natural Resources and Life Sciences (BOKU), Muthgasse 18, 1190 Vienna, Austria

[4] Cardiff School of Biosciences, The Sir Martin Evans Building, Museum Avenue, Cardiff, CF10 3AX, United Kingdom

[*]Correspondence: jiri.friml@ist.ac.at

Fax: +43 (0)2243 9000 2000

Supplementary Figure 1. Morphological analysis of lines expressing pin3 phospho-mutant variants. (**a**) Quantification of root bending after 12 hours gravistimulation of different d6pk mutant and overexpression lines. (**b**) qPCR evaluation of PIN3 transcript levels in transformed plants (*pin3-4*). (**c-g**) Phenotypic analysis of PIN3 mutant variants expressing lines: root length (c); root meristem size (d); emerged lateral root number (e) and lateral root stages (f) and hypocotyl length of dark grown seedlings (g). Experiments were repeated 3 times with 10-15 roots/hypocotyls per line. Student's T-tests were calculated for the comparison of each line with the control (Col-0). Error bars represent SE, (* $p<0.05$, ** $p<0.01$, *** $p<0.001$).

Supplementary Figure 2. Lane profiles from anti-GFP blots and PIN3-YFP localization in hypocotyl. (**a**) Lane profiles of the anti-GFP blots for different PIN3-YFP mutant variants with or without PINOID from Fig. 3a. Phosphorylation is seen as additional bump (marked by arrow) migrating slower than the main peak. PIN3-YFP before and after 4 hours of gravitropic stimulation in wild type (**b**); and before gravitropic stimulation in PIN3-YFP-P1A (**c**), PIN3-YFP-P1D (**d**), PIN3-YFP-P2A (**e**), and PIN3-YFP-P2D (**f**). Yellow arrows indicate gravity vector. Bars = 10 µm.

Supplementary Figure 3. Uncropped blots from *in vivo* phosphorylation of PIN3-YFP and mutant variants. PIN3-YFP and mutant variants were transiently expressed in *N. benthamiana* with or without co-infiltration of PINOID. (**a**) Complete blots, various exposures and Ponceau stain for Figure 3a. Despite equivalent protein loading, various exposures for anti-GFP were required due to different expression levels of PIN3-YFP and mutant variants. Longer exposures were required for PIN3-YFP-P2A + PINOID and PIN3-YFP-P2D + PINOID (right hand panels, anti-GFP). Breakdown products are indicated. As *N. benthamiana* leaves contain a strong non-specific signal from anti-FLAG antibody at about 90 kD, only the lower half of the blot was used for anti-FLAG. The anti-FLAG signal for PIN3-YFP-P2D + PINOID was too weak to be detected on this blot; the same samples were re-run on a different blot (right panel, anti-FLAG). N represents carryover signal from anti-GFP (breakdown product of PIN3-YFP-P1D). (**b**) Complete blot and Ponceau staining of anti-GFP for Figures 3c. (**c**) Complete blot and Ponceau staining of Phostag gel for Figures 3d.

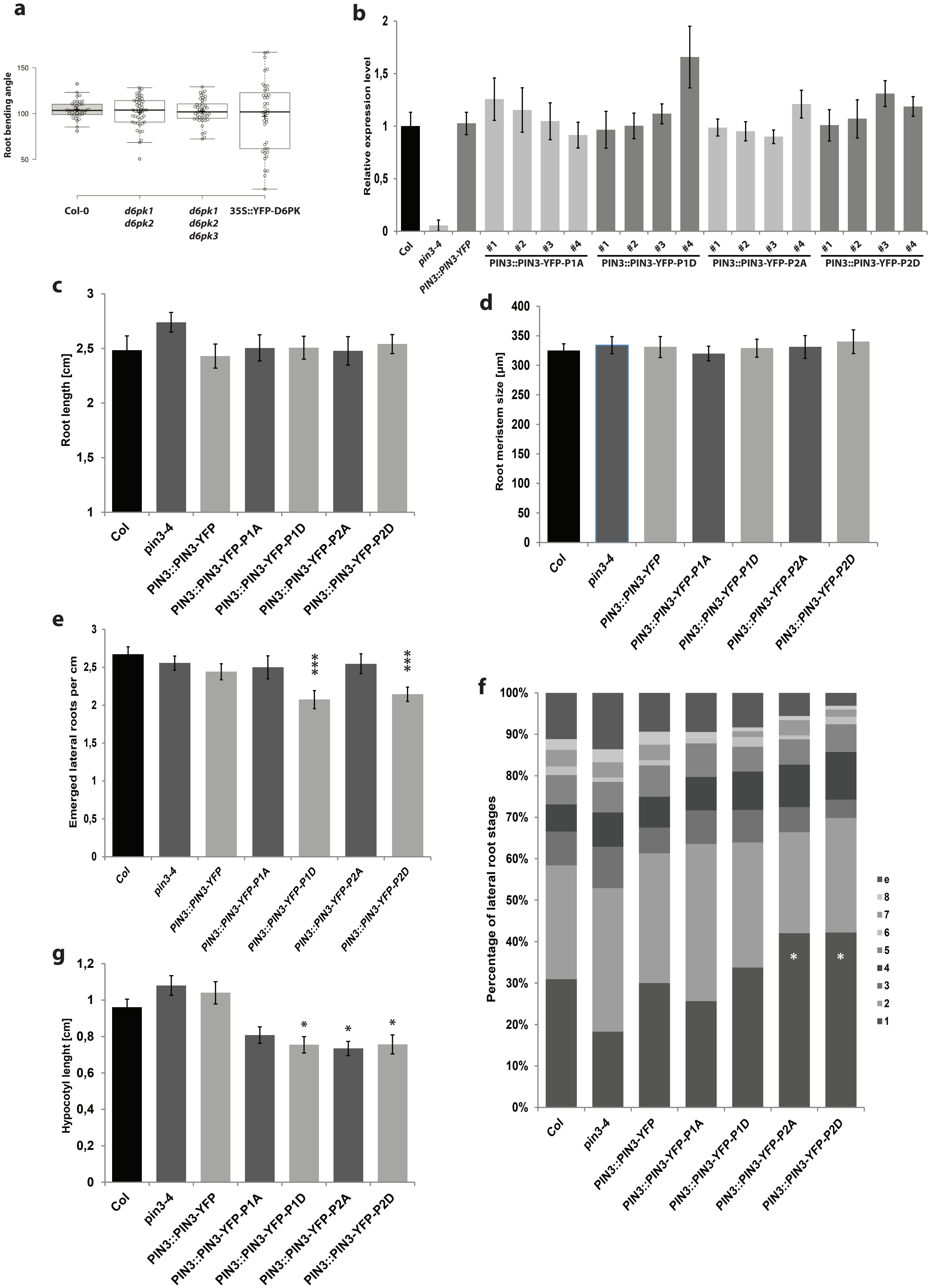

a
Root bending angle
150
100
50
Col-0
d6pk1 d6pk2
d6pk1 d6pk2 d6pk3
35S::YFP-D6PK
b
Relative expression level
2
1,5
1
0,5
0
Col
pin3-4
PIN3::PIN3-YFP
#1 #2 #3 #4
PIN3::PIN3-YFP-P1A
PIN3::PIN3-YFP-P1D
PIN3::PIN3-YFP-P2A
PIN3::PIN3-YFP-P2D
c
Root length [cm]
3
2,5
2
1,5
1
d
Root meristem size [µm]
400
350
300
250
200
150
100
50
0
e
Emerged lateral roots per cm
3
2,5
2
1,5
1
0,5
0
***
***
f
Percentage of lateral root stages
100%
90%
80%
70%
60%
50%
40%
30%
20%
10%
0%
*
*
e
8
7
6
5
4
3
2
1
g
Hypocotyl lenght [cm]
1,2
1
0,8
0,6
0,4
0,2
0
*
*
*
Col
pin3-4
PIN3::PIN3-YFP
PIN3::PIN3-YFP-P1A
PIN3::PIN3-YFP-P1D
PIN3::PIN3-YFP-P2A
PIN3::PIN3-YFP-P2D

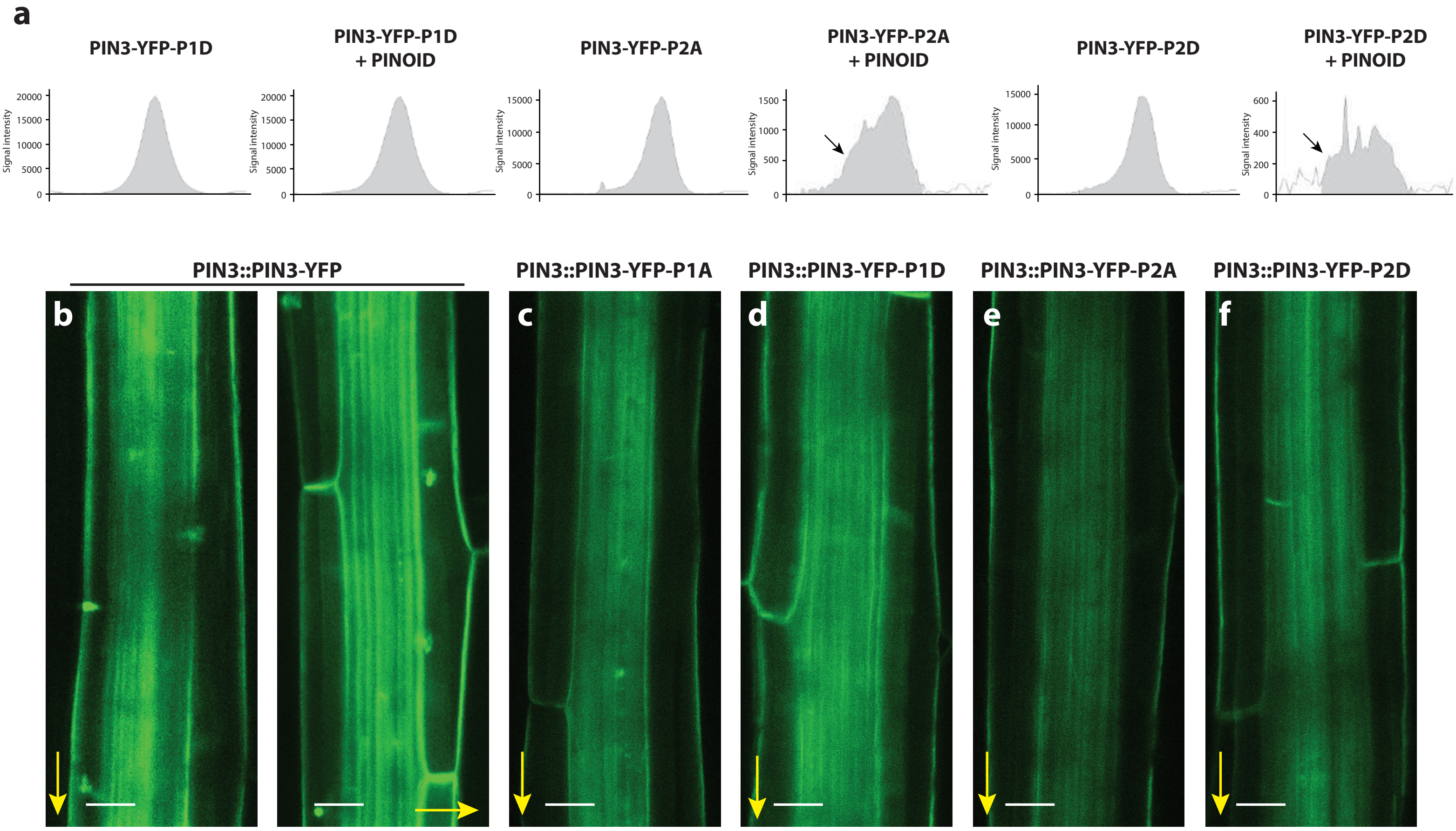
a
PIN3-YFP-P1D
PIN3-YFP-P1D + PINOID
PIN3-YFP-P2A
PIN3-YFP-P2A + PINOID
PIN3-YFP-P2D
PIN3-YFP-P2D + PINOID
Signal intensity
PIN3::PIN3-YFP
PIN3::PIN3-YFP-P1A
PIN3::PIN3-YFP-P1D
PIN3::PIN3-YFP-P2A
PIN3::PIN3-YFP-P2D
b
c
d
e
f

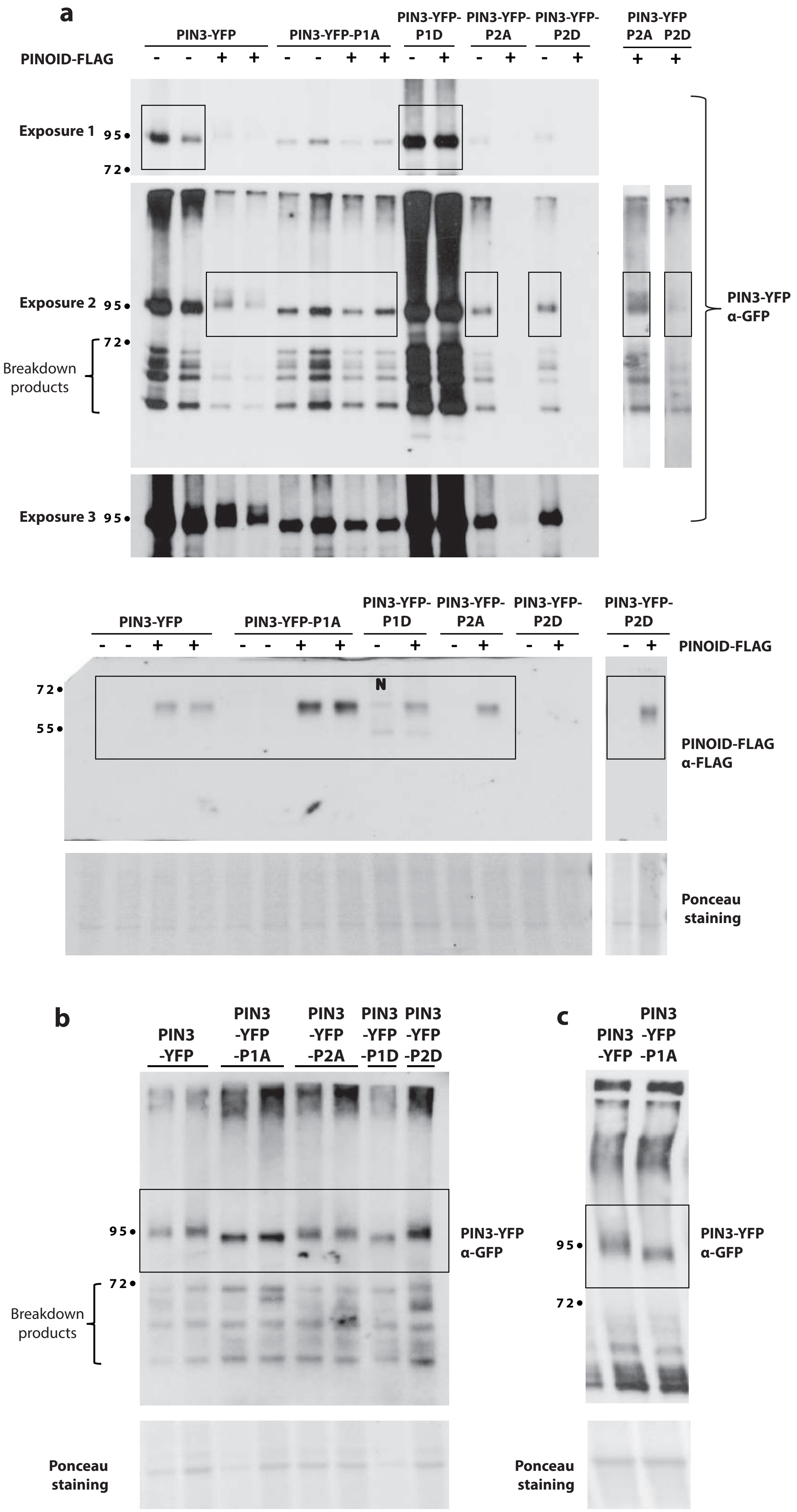

a
PIN3-YFP
PIN3-YFP-P1A
PIN3-YFP-P1D
PIN3-YFP-P2A
PIN3-YFP-P2D
PIN3-YFP P2A P2D
PINOID-FLAG
- - + + - - + + - + - + - + + +
Exposure 1
95
72
Exposure 2
95
72
Breakdown products
Exposure 3
95
PIN3-YFP α-GFP
PIN3-YFP
PIN3-YFP-P1A
PIN3-YFP-P1D
PIN3-YFP-P2A
PIN3-YFP-P2D
PIN3-YFP-P2D
- - + + - - + + - + - + - + - +
PINOID-FLAG
72
55
N
PINOID-FLAG α-FLAG
Ponceau staining
b
PIN3 -YFP
PIN3 -YFP -P1A
PIN3 -YFP -P2A
PIN3 -YFP -P1D
PIN3 -YFP -P2D
95
72
PIN3-YFP α-GFP
Breakdown products
Ponceau staining
c
PIN3 -YFP
PIN3 -YFP -P1A
95
72
PIN3-YFP α-GFP
Ponceau staining

**PIN3 -P1A**	CTCGAGTGGAGCATCACAATCTTTTCCCTCTCCACACTTCCCAACACTCTTGTTATGGGGATTCCTCTCTTGATCGCCA<br>TGTATGGCGAATACTCTGGTTCCCTCATGGTCCAAATCGTCGTCCTCCAGTGTATCATCTGGTACACGCTTCTCCTTTT<br>TCTCTTCGAGTTTCGTGGCGCCAAGATGCTCATCATGGAGCAGTTCCCTGAGACGGCTGCTTCCATTGTTTCTTTCAAA<br>GTCGAATCCGACGTCGTTTCGCTCGACGGCCATGATTTTCTTGAGACCGATGCAGAGATAGGTGACGACGGGAAGCTTC<br>ACGTCACCGTGAGAAAATCCAACGCTTCACGTCGTTCGTTCTGCGGCCCGAACATGACTCCACGGCCGGCAAATCTCAC<br>CGGAGCTGAGATTTATAGTCTCAGCACCACTCCTAGAGGCGCTAATTTCAACCACTCTGATTTTTACAACATGATGGGT<br>TTCCCCGGTGGTCGTCTCTCCAATTTCGGTCCGGCGGATATGTACTCCGTTCAATCATCTAGAGGTCCAACTCCTCGAC<br>CTGCAAACTTCGAGGAGAATTGCGCCATGGCATCCTCCCCGAGATTCGGGTATTACCCTGGAGGAGGAGCCGGGTCTTA<br>TCCGGCTCCGAATCCAGAGTTCTCTTCAACCACCACATCTACCGCCAATAAAAGCGTCAATAAAAACCCGAAAGACGTT<br>AATACGAATCAGCAGACGACTCTTCCAACGGGCGGCAAGTCAAACAGCCATGACGCCAAGGAGCTTCACATGTTCGTCT<br>GGAGCTCAAACGGGTCACCCGTTTCAGACCGGGCGGGTCTTAACGTTTTCGGCGGAGCACCTGACAACGATCAAGGCGG<br>AAGATCTGACCAAGGTGCTAAAGAGATCCGTATGTTAGTCCCAGATCAATCTCACAACGGCGAGACCAAAGGGGGTGGC<br>ATGGTGAGCAAGGGCGAGGAGCTGTTCACCGGGGTGGTGCCCATCCTGGTCGAGCTGGACGGCGACGTAAACGGCCACA<br>AGTTCAGCGTGTCCGGCGAGGGCGAGGGCGATGCCACCTACGGCAAGCTGACCCTGAAGCTGATCTGCACCACCGGCAA<br>GCTGCCCGTGCCCTGGCCCACCCTCGTGACCACCCTGGGCTACGGCCTGCAGTGCTTCGCCCGCTACCCCGACCACATG<br>AAGCAGCACGACTTCTTCAAGTCCGCCATGCCCGAAGGCTACGTCCAGGAGCGCACCATCTTCTTCAAGGACGACGGCA<br>ACTACAAGACCCGCGCCGAGGTGAAGTTCGAGGGCGACACCCTGGTGAACCGCATCGAGCTGAAGGGCATCGACTTCAA<br>GGAGGACGGCAACATCCTGGGGCACAAGCTGGAGTACAACTACAACAGCCACAACGTCTATATCACCGCCGACAAGCAG<br>AAGAACGGCATCAAGGCCAACTTCAAGATCCGCCACAACATCGAGGACGGCGGCGTGCAGCTCGCCGACCACTACCAGC<br>AGAACACCCCCATCGGCGACGGCCCCGTGCTGCTGCCCGACAACCACTACCTGAGCTACCAGTCCGCCCTGAGCAAAGA<br>CCCCAACGAGAAGCGCGATCACATGGTCCTGCTGGAGTTCGTGACCGCCGCCGGGATCACTCTCGGCATGGACGAGCTG<br>TACAAGGGAGGTGGACCCGGTTAGCAATCTTTAGATAACTTTCTTGATAAGTCACTGCAAATTTTCTCAATTCTGTTTT<br>GTGTGGTGAATATAGCTGTAGCTCATCCAGCAAGTGGAGATTTCGGAGGAGAACAACAATTTAGTTTCGCCGGAAAAGA<br>AGAAGAAGCAGAGAGACCAAAAGACGCCGAGAATGGTCTAAACAAACTTGCTCCAAATTCCACGGCGGCGCTACAATCC<br>AAGACAGGTCTAGGAGGAGCCGAAGCAAGTCAACGAAAAAATATGCCTCCGGCGAGTGTGATGACAAGGCTGATACTGA<br>TAATGGTTTGGAGGAAACTCATCAGAAACCCAAACACTTACTCTAGTCTCATTGGACTTATTTGGGCTCTCGTCGCTTT<br>CCGGTTAGTAAATCAAAATTAATTGTTTTCTTAACTCGAACCAACCGGTT
**PIN3 -P1D**	CTCGAGTGGAGCATCACAATCTTTTCCCTCTCCACACTTCCCAACACTCTTGTTATGGGGATTCCTCTCTTGATCGCCA<br>TGTATGGCGAATACTCTGGTTCCCTCATGGTCCAAATCGTCGTCCTCCAGTGTATCATCTGGTACACGCTTCTCCTTTT<br>TCTCTTCGAGTTTCGTGGCGCCAAGATGCTCATCATGGAGCAGTTCCCTGAGACGGCTGCTTCCATTGTTTCTTTCAAA<br>GTCGAATCCGACGTCGTTTCGCTCGACGGCCATGATTTTCTTGAGACCGATGCAGAGATAGGTGACGACGGGAAGCTTC<br>ACGTCACCGTGAGAAAATCCAACGCTTCACGTCGTTCGTTCTGCGGCCCGAACATGACTCCACGGCCGGATAATCTCAC<br>CGGAGCTGAGATTTATAGTCTCAGCACCACTCCTAGAGGCGATAATTTCAACCACTCTGATTTTTACAACATGATGGGT<br>TTCCCCGGTGGTCGTCTCTCCAATTTCGGTCCGGCGGATATGTACTCCGTTCAATCATCTAGAGGTCCAACTCCTCGAC<br>CTGATAACTTCGAGGAGAATTGCGCCATGGCATCCTCCCCGAGATTCGGGTATTACCCTGGAGGAGGAGCCGGGTCTTA<br>TCCGGCTCCGAATCCAGAGTTCTCTTCAACCACCACATCTACCGCCAATAAAAGCGTCAATAAAAACCCGAAAGACGTT<br>AATACGAATCAGCAGACGACTCTTCCAACGGGCGGCAAGTCAAACAGCCATGACGCCAAGGAGCTTCACATGTTCGTCT<br>GGAGCTCAAACGGGTCACCCGTTTCAGACCGGGCGGGTCTTAACGTTTTCGGCGGAGCACCTGACAACGATCAAGGCGG<br>AAGATCTGACCAAGGTGCTAAAGAGATCCGTATGTTAGTCCCAGATCAATCTCACAACGGCGAGACCAAAGGGGGTGGC<br>ATGGTGAGCAAGGGCGAGGAGCTGTTCACCGGGGTGGTGCCCATCCTGGTCGAGCTGGACGGCGACGTAAACGGCCACA<br>AGTTCAGCGTGTCCGGCGAGGGCGAGGGCGATGCCACCTACGGCAAGCTGACCCTGAAGCTGATCTGCACCACCGGCAA<br>GCTGCCCGTGCCCTGGCCCACCCTCGTGACCACCCTGGGCTACGGCCTGCAGTGCTTCGCCCGCTACCCCGACCACATG<br>AAGCAGCACGACTTCTTCAAGTCCGCCATGCCCGAAGGCTACGTCCAGGAGCGCACCATCTTCTTCAAGGACGACGGCA<br>ACTACAAGACCCGCGCCGAGGTGAAGTTCGAGGGCGACACCCTGGTGAACCGCATCGAGCTGAAGGGCATCGACTTCAA<br>GGAGGACGGCAACATCCTGGGGCACAAGCTGGAGTACAACTACAACAGCCACAACGTCTATATCACCGCCGACAAGCAG<br>AAGAACGGCATCAAGGCCAACTTCAAGATCCGCCACAACATCGAGGACGGCGGCGTGCAGCTCGCCGACCACTACCAGC<br>AGAACACCCCCATCGGCGACGGCCCCGTGCTGCTGCCCGACAACCACTACCTGAGCTACCAGTCCGCCCTGAGCAAAGA<br>CCCCAACGAGAAGCGCGATCACATGGTCCTGCTGGAGTTCGTGACCGCCGCCGGGATCACTCTCGGCATGGACGAGCTG<br>TACAAGGGAGGTGGACCCGGTTAGCAATCTTTAGATAACTTTCTTGATAAGTCACTGCAAATTTTCTCAATTCTGTTTT<br>GTGTGGTGAATATAGCTGTAGCTCATCCAGCAAGTGGAGATTTCGGAGGAGAACAACAATTTAGTTTCGCCGGAAAAGA<br>AGAAGAAGCAGAGAGACCAAAAGACGCCGAGAATGGTCTAAACAAACTTGCTCCAAATTCCACGGCGGCGCTACAATCC<br>AAGACAGGTCTAGGAGGAGCCGAAGCAAGTCAACGAAAAAATATGCCTCCGGCGAGTGTGATGACAAGGCTGATACTGA<br>TAATGGTTTGGAGGAAACTCATCAGAAACCCAAACACTTACTCTAGTCTCATTGGACTTATTTGGGCTCTCGTCGCTTT<br>CCGGTTAGTAAATCAAAATTAATTGTTTTCTTAACTCGAACCAACCGGTT

**PIN3 -P2A**	CTCGAGTGGAGCATCACAATCTTTTCCCTCTCCACACTTCCCAACACTCTTGTTATGGGGATTCCTCTCTTGATCGCCA<br>TGTATGGCGAATACTCTGGTTCCCTCATGGTCCAAATCGTCGTCCTCCAGTGTATCATCTGGTACACGCTTCTCCTTTT<br>TCTCTTCGAGTTTCGTGGCGCCAAGATGCTCATCATGGAGCAGTTCCCTGAGACGGCTGCTTCCATTGTTTCTTTCAAA<br>GTCGAATCCGACGTCGTTTCGCTCGACGGCCATGATTTTCTTGAGACCGATGCAGAGATAGGTGACGACGGGAAGCTTC<br>ACGTCACCGTGAGAAAATCCAACGCTTCACGTCGTTCGTTCTGCGGCCCGAACATGACTCCACGGCCGTCAAATCTCAC<br>CGGAGCTGAGATTTATAGTCTCAGCACCACTCCTAGAGGCTCTAATTTCAACCACTCTGATTTTTACAACATGATGGGT<br>TTCCCCGGTGGTCGTCTCTCCAATTTCGGTCCGGCGGATATGTACTCCGTTCAATCATCTAGAGGTCCAACTCCTCGAC<br>CTTCAAACTTCGAGGAGAATTGCGCCATGGCATCCTCCCCGAGATTCGGGTATTACCCTGGAGGAGGAGCCGGGTCTTA<br>TCCGGCTCCGAATCCAGAGTTCGCTGCAACCACCACAGCTACCGCCAATAAAAGCGTCAATAAAAACCCGAAAGACGTT<br>AATACGAATCAGCAGACGACTCTTCCAACGGGCGGCAAGTCAAACAGCCATGACGCCAAGGAGCTTCACATGTTCGTCT<br>GGAGCTCAAACGGGTCACCCGTTTCAGACCGGGCGGGTCTTAACGTTTTCGGCGGAGCACCTGACAACGATCAAGGCGG<br>AAGATCTGACCAAGGTGCTAAAGAGATCCGTATGTTAGTCCCAGATCAATCTCACAACGGCGAGACCAAAGGGGGTGGC<br>ATGGTGAGCAAGGGCGAGGAGCTGTTCACCGGGGTGGTGCCCATCCTGGTCGAGCTGGACGGCGACGTAAACGGCCACA<br>AGTTCAGCGTGTCCGGCGAGGGCGAGGGCGATGCCACCTACGGCAAGCTGACCCTGAAGCTGATCTGCACCACCGGCAA<br>GCTGCCCGTGCCCTGGCCCACCCTCGTGACCACCCTGGGCTACGGCCTGCAGTGCTTCGCCCGCTACCCCGACCACATG<br>AAGCAGCACGACTTCTTCAAGTCCGCCATGCCCGAAGGCTACGTCCAGGAGCGCACCATCTTCTTCAAGGACGACGGCA<br>ACTACAAGACCCGCGCCGAGGTGAAGTTCGAGGGCGACACCCTGGTGAACCGCATCGAGCTGAAGGGCATCGACTTCAA<br>GGAGGACGGCAACATCCTGGGGCACAAGCTGGAGTACAACTACAACAGCCACAACGTCTATATCACCGCCGACAAGCAG<br>AAGAACGGCATCAAGGCCAACTTCAAGATCCGCCACAACATCGAGGACGGCGGCGTGCAGCTCGCCGACCACTACCAGC<br>AGAACACCCCCATCGGCGACGGCCCCGTGCTGCTGCCCGACAACCACTACCTGAGCTACCAGTCCGCCCTGAGCAAAGA<br>CCCCAACGAGAAGCGCGATCACATGGTCCTGCTGGAGTTCGTGACCGCCGCCGGGATCACTCTCGGCATGGACGAGCTG<br>TACAAGGGAGGTGGACCCGGTTAGCAATCTTTAGATAACTTTCTTGATAAGTCACTGCAAATTTTCTCAATTCTGTTTT<br>GTGTGGTGAATATAGCTGTAGCTCATCCAGCAAGTGGAGATTTCGGAGGAGAACAACAATTTAGTTTCGCCGGAAAAGA<br>AGAAGAAGCAGAGAGACCAAAAGACGCCGAGAATGGTCTAAACAAACTTGCTCCAAATTCCACGGCGGCGCTACAATCC<br>AAGACAGGTCTAGGAGGAGCCGAAGCAAGTCAACGAAAAAATATGCCTCCGGCGAGTGTGATGACAAGGCTGATACTGA<br>TAATGGTTTGGAGGAAACTCATCAGAAACCCAAACACTTACTCTAGTCTCATTGGACTTATTTGGGCTCTCGTCGCTTT<br>CCGGTTAGTAAATCAAAATTAATTGTTTTCTTAACTCGAACCAACCGGTT
**PIN3 -P2D**	CTCGAGTGGAGCATCACAATCTTTTCCCTCTCCACACTTCCCAACACTCTTGTTATGGGGATTCCTCTCTTGATCGCCA<br>TGTATGGCGAATACTCTGGTTCCCTCATGGTCCAAATCGTCGTCCTCCAGTGTATCATCTGGTACACGCTTCTCCTTTT<br>TCTCTTCGAGTTTCGTGGCGCCAAGATGCTCATCATGGAGCAGTTCCCTGAGACGGCTGCTTCCATTGTTTCTTTCAAA<br>GTCGAATCCGACGTCGTTTCGCTCGACGGCCATGATTTTCTTGAGACCGATGCAGAGATAGGTGACGACGGGAAGCTTC<br>ACGTCACCGTGAGAAAATCCAACGCTTCACGTCGTTCGTTCTGCGGCCCGAACATGACTCCACGGCCGTCAAATCTCAC<br>CGGAGCTGAGATTTATAGTCTCAGCACCACTCCTAGAGGCTCTAATTTCAACCACTCTGATTTTTACAACATGATGGGT<br>TTCCCCGGTGGTCGTCTCTCCAATTTCGGTCCGGCGGATATGTACTCCGTTCAATCATCTAGAGGTCCAACTCCTCGAC<br>CTTCAAACTTCGAGGAGAATTGCGCCATGGCATCCTCCCCGAGATTCGGGTATTACCCTGGAGGAGGAGCCGGGTCTTA<br>TCCGGCTCCGAATCCAGAGTTCGATGATACCACCACAGATACCGCCAATAAAAGCGTCAATAAAAACCCGAAAGACGTT<br>AATACGAATCAGCAGACGACTCTTCCAACGGGCGGCAAGTCAAACAGCCATGACGCCAAGGAGCTTCACATGTTCGTCT<br>GGAGCTCAAACGGGTCACCCGTTTCAGACCGGGCGGGTCTTAACGTTTTCGGCGGAGCACCTGACAACGATCAAGGCGG<br>AAGATCTGACCAAGGTGCTAAAGAGATCCGTATGTTAGTCCCAGATCAATCTCACAACGGCGAGACCAAAGGGGGTGGC<br>ATGGTGAGCAAGGGCGAGGAGCTGTTCACCGGGGTGGTGCCCATCCTGGTCGAGCTGGACGGCGACGTAAACGGCCACA<br>AGTTCAGCGTGTCCGGCGAGGGCGAGGGCGATGCCACCTACGGCAAGCTGACCCTGAAGCTGATCTGCACCACCGGCAA<br>GCTGCCCGTGCCCTGGCCCACCCTCGTGACCACCCTGGGCTACGGCCTGCAGTGCTTCGCCCGCTACCCCGACCACATG<br>AAGCAGCACGACTTCTTCAAGTCCGCCATGCCCGAAGGCTACGTCCAGGAGCGCACCATCTTCTTCAAGGACGACGGCA<br>ACTACAAGACCCGCGCCGAGGTGAAGTTCGAGGGCGACACCCTGGTGAACCGCATCGAGCTGAAGGGCATCGACTTCAA<br>GGAGGACGGCAACATCCTGGGGCACAAGCTGGAGTACAACTACAACAGCCACAACGTCTATATCACCGCCGACAAGCAG<br>AAGAACGGCATCAAGGCCAACTTCAAGATCCGCCACAACATCGAGGACGGCGGCGTGCAGCTCGCCGACCACTACCAGC<br>AGAACACCCCCATCGGCGACGGCCCCGTGCTGCTGCCCGACAACCACTACCTGAGCTACCAGTCCGCCCTGAGCAAAGA<br>CCCCAACGAGAAGCGCGATCACATGGTCCTGCTGGAGTTCGTGACCGCCGCCGGGATCACTCTCGGCATGGACGAGCTG<br>TACAAGGGAGGTGGACCCGGTTAGCAATCTTTAGATAACTTTCTTGATAAGTCACTGCAAATTTTCTCAATTCTGTTTT<br>GTGTGGTGAATATAGCTGTAGCTCATCCAGCAAGTGGAGATTTCGGAGGAGAACAACAATTTAGTTTCGCCGGAAAAGA<br>AGAAGAAGCAGAGAGACCAAAAGACGCCGAGAATGGTCTAAACAAACTTGCTCCAAATTCCACGGCGGCGCTACAATCC<br>AAGACAGGTCTAGGAGGAGCCGAAGCAAGTCAACGAAAAAATATGCCTCCGGCGAGTGTGATGACAAGGCTGATACTGA<br>TAATGGTTTGGAGGAAACTCATCAGAAACCCAAACACTTACTCTAGTCTCATTGGACTTATTTGGGCTCTCGTCGCTTT<br>CCGGTTAGTAAATCAAAATTAATTGTTTTCTTAACTCGAACCAACCGGTT

Table S1. Sequences of synthesized fragments containing the PIN3 phosphhorylation mutations.
